# An IL-6-IL-8 score derived from principal component analysis is predictive of adverse outcome in acute myocardial infarction

**DOI:** 10.1016/j.cytox.2020.100037

**Published:** 2020-10-08

**Authors:** Gisela A. Kristono, Ana S. Holley, Kathryn E. Hally, Morgane M. Brunton-O'Sullivan, Bijia Shi, Scott A. Harding, Peter D. Larsen

**Affiliations:** a Department of Surgery and Anaesthesia, University of Otago Wellington, New Zealand; b Wellington Cardiovascular Research Group, New Zealand; c School of Biological Sciences, Victoria University of Wellington, New Zealand; d Cardiology Department, Capital and Coast District Health Board, New Zealand

**Keywords:** Cytokine score, Principal component analysis, Interleukin-6, Interleukin-8, Acute myocardial infarction, Major adverse cardiovascular events, ACS, Acute coronary syndrome, AF, Atrial fibrillation, AMI, Acute myocardial infarction, AUC, Area under the curve, BMI, Body mass index, CAD, Coronary artery disease, CBA, Cytometric bead array, CHF, Chronic heart failure, CI, Confidence interval, CVD, Cardiovascular disease, EFA, Exploratory factor analysis, ELISA, Enzyme-linked immunosorbent assay, GDF-15, Growth differentiation factor-15, GM-CSF, Granulocyte-macrophage colony-stimulating factor, h, Hours, HTN, Hypertension, IFNγ, Interferon gamma, IL-(number), Interleukin-(number), IQR, Interquartile range, MCP-1, Monocyte chemoattractant protein-1, MFI, Mean fluorescence intensity, MACE, Major adverse cardiovascular events, MI, Myocardial infarction, NSTEMI, Non-ST elevation myocardial infarction, OR, Odds ratio, p, P-value, PCA, Principal component analysis, PCI, Percutaneous coronary intervention, RANTES, Regulated upon activation normal T-cell expressed and secreted, ROC, Receiver operator characteristic, STEMI, ST-elevation myocardial infarction, TGF-β1, Tumour growth factor-beta 1, TIA, Transient ischaemic attack, TNF-α, Tumour necrosis factor alpha, TnT, Troponin T, TRAIL-R2, Tumour necrosis factor-related apoptosis-inducing ligand receptor 2, VEGF, Vascular endothelial growth factor

## Abstract

**Introduction:**

Many studies have shown that elevated biomarkers of inflammation following acute myocardial infarction (AMI) are associated with major adverse cardiovascular events (MACE). However, the optimal way of measuring the complex inflammatory response following AMI has not been determined. In this study we explore the use of principal component analysis (PCA) utilising multiple inflammatory cytokines to generate a combined cytokine score that may be predictive of MACE post-AMI.

**Methods:**

Thirteen inflammatory cytokines were measured in plasma of 317 AMI patients, drawn 48–72 h following symptom onset. Patients were followed-up for one year to determine the incidence of MACE. PCA was used to generate a combined score using six cytokines that were detectable in the majority of patients (IL-1β, -6, -8, and -10; MCP-1; and RANTES), and using a subset of cytokines that were associated with MACE on univariate analysis. Multivariate models using baseline characteristics, elevated individual cytokines and PCA-derived scores determined independent predictors of MACE.

**Results:**

IL-6 and IL-8 were significantly associated with MACE on univariate analysis and were combined using PCA into an IL-6-IL-8 score. The combined cytokine score and IL-6-IL-8 PCA-derived score were both significantly associated with MACE on univariate analysis. In multivariate models IL-6-IL-8 scores (OR = 2.77, p = 0.007) and IL-6 levels (OR = 2.18, p = 0.035) were found to be independent predictors of MACE.

**Conclusion:**

An IL-6-IL-8 score derived from PCA was found to independently predict MACE at one year and was a stronger predictor than any individual cytokine, which suggests this may be an appropriate strategy to quantify inflammation post-AMI. Further investigation is required to determine the optimal set of cytokines to measure in this context.

## Highlights

• Principal component analysis (PCA) can create scores from collinear markers.
• This study shows a PCA-derived score can combine cytokines in AMI patients.
• An IL-6-IL-8 score using PCA independently predicts poor outcomes in AMI.

## 1 Introduction

Globally, cardiovascular disease (CVD) is a leading cause of death, with acute myocardial infarction (AMI) being a significant contributor to CVD burden [1]. Inflammation plays a critical role in the process of atherosclerosis that leads to AMI, and in the resolution and healing that occurs after infarction [2]. However, suboptimal levels of inflammation can have detrimental effects on this repair process [2]. Cytokines are an important subset of inflammatory markers and their elevated levels have been associated with adverse remodelling and outcomes after AMI [3], [4].

Traditionally, studies have focussed on the association between single cytokines and major adverse cardiovascular events (MACE) following AMI. However, there has been increasing recognition that inflammation following AMI is a complex process and investigating multiple cytokines using a combined analytical method may be beneficial [5], [6], [7], [8], [9]. The methods used to date have been relatively simple, such as combining a pro- and an anti-inflammatory cytokine into a ratio or adding a point to a patient’s inflammatory score for every cytokine elevated above the median [5], [6], [7]. Unfortunately, using ratios limits the combination to only two cytokines that have assumed opposing actions. These methods also assume that cytokines are independent factors, which inherently places a greater weighting on correlated cytokines with overlapping functions, causing them to be over-represented in the derived score. There is a need to create a combined cytokine score using a mathematical approach that can counter these flaws.

One mathematical approach is principal component analysis (PCA). PCA is a statistical method that reduces complex, multi-dimensional data while retaining maximal variance [10]. It makes no assumptions on the independence between variables, which means multi-collinear data can be combined into scores. Other benefits are that PCA can be conducted with small portions of missing data for individual patients and that, by reducing the number of variables, a smaller cohort size can be used to measure outcomes using multivariate analysis in a statistically-meaningful way [11]. PCA has been used in studies looking at adverse outcome in conditions such as subarachnoid haemorrhage and trauma, where the authors derived prognostic scores from the principal components and found these to be predictive of outcome [12], [13].

The primary aim of this study was to create a PCA-derived score composed of cytokines associated with inflammation in AMI patients, and assess its association with major adverse cardiovascular events (MACE).

## 2 Methods

### 2.1 Study population

All patients that were included in this observational cohort study were selected from an existing AMI biobank. Patients were recruited into this biobank if they were admitted to Wellington Regional Hospital, New Zealand, between January 2012 and April 2018 with a diagnosis of spontaneous (type 1) AMI and were planned to be treated with an invasive approach (angiography with or without revascularisation). AMI was defined according to the third universal definition [14]. Exclusion criteria included types 2–5 myocardial infarction (MI), chronic inflammatory disorders, malignancy, and immune-modulating medications. All patients gave voluntary written consent for their participation in this biobank and the study was approved by the New Zealand Lower South Ethics Committee (LRS/11/09/035) and the New Zealand Central Health and Disability Ethics Committee (16/CEN/68).

From this population, patients were selected for this study if they had not been treated with an anti-thrombolytic agent, had no renal insufficiency and had plasma samples collected between 48 and 72 h after onset of their ischemic symptoms.

The primary endpoint was defined as MACE at one year post index admission with AMI. MACE was defined hierarchically as a composite of all-cause death, non-fatal MI, non-fatal ischaemic cerebrovascular accident, stent thrombosis, chronic heart failure (CHF) leading to hospital admission, and unplanned revascularisation. Stent thrombosis included either probable or definite stent thrombosis as defined according to the Academic Research Consortium criteria [15].

### 2.2 Data collection and sampling methods

Baseline demographics and clinical information were prospectively collected using hospital records and the cardiac catheterisation database. Follow-up was conducted by research nurses at 12 months through telephone calls, hospital electronic records, and where necessary, contacting participants’ family doctors.

Between 48 and 72 h from symptom onset, whole blood samples were collected in sodium citrate tubes (0.109 M BD Vacutainer, New Jersey, USA) from the peripheral vein prior to cardiac catheterisation or from the arterial sheath immediately after insertion and before administration of heparin during coronary angiography. The tubes were centrifuged within 60 min of collection at 1500*g* for 12 min at room temperature. After centrifugation, the separated plasma was stored at −80 °C until analysis.

### 2.3 Cytokine analysis

A total of 13 cytokines were analysed: Interleukin (IL) -1β, IL-4, IL-6, IL-8, IL-10, IL-17A, granulocyte–macrophage colony-stimulating factor (GM-CSF), interferon gamma (IFN-γ), monocyte chemoattractant protein-1 (MCP-1), regulated upon activation normal T cell expressed and secreted (RANTES), transforming growth factor-beta 1 (TGF-β1), tumour necrosis factor alpha (TNFα), and vascular endothelial growth factor (VEGF). These cytokines were analysed using cytometric bead array (CBA) and enzyme-linked immunosorbent assay (ELISA) kits. Supplementary Table 1 presents the rationales for the chosen cytokines and the concentrations of the lowest standards for each cytokine (defined by the manufacturer). The CBA kits were multiplexed in the following configurations: Panel One consisted of seven cytokines, IL-4, IL-6, IL-8, IL-10, IL-17A, IFN-γ and TNFα (Enhanced Sensitivity Human Soluble Protein CBA Flex Set, BD Biosciences; CA, USA); Panel Two consisted of four cytokines, GM-CSF, MCP-1, RANTES and VEGF (Human Soluble Protein CBA, BD Biosciences; CA, USA); and Panel Three consisted of TGF-β1 only, analysed as a single plex (Human Soluble Protein CBA Flex Set, BD Biosciences; CA, USA). All samples were analysed on a BD FACSCanto II flow cytometer (BD Biosciences; CA, USA). A commercially available ELISA kit was used to quantify levels of IL-1β in the plasma (Human IL-1β Ready-Set-Go ELISA, Invitrogen, Thermo Fisher Scientific; CA, USA). The analyses were conducted according to the manufacturers’ instructions.

### 2.4 Statistical analysis

For the CBA plates, data collected from the flow cytometer was analysed on FlowJo® (FlowJo, LLC; OR, USA) and FCAP Array Software v3.0 (BD Biosciences; CA USA) to obtain a mean fluorescence intensity (MFI). The concentrations of each cytokine were calculated using a 5PL fitted standard curve. Samples values below the blank were defined as “undetectable” and given a concentration of 0 pg/mL. Samples that had MFIs or absorbances above the blank but below the lowest standard (i.e. undetermined concentrations when fitted with the 5PL curve) were extrapolated using linear regression down to zero. Samples with MFIs for RANTES above the upper limit of detection were all given one concentration that was greater than the highest detected concentration, and this was arbitrarily chosen as 16,000 pg/mL. Unless specified, analysis was conducted on GraphPad Prism v.7 (GraphPad Software Inc.; CA, USA).

Descriptive statistics for baseline demographics and cytokine concentrations, tests of normality (Shapiro-Wilk and D’Agostino and Pearson) for the cytokine concentrations and baseline demographics were analysed on GraphPad Prism v.7. Cytokines where over half of the patients had detectable concentrations were used to create a combined cytokine score by the addition of significant component scores derived from principal component analysis (PCA). Parallel analysis was used to determine the number of significant components. Regression component scores were generated without rotation and the component matrix was used to observe which cytokines contributed significantly to the loading of each component score (correlation > 0.5). Finally, the component scores were added to create the cytokine score. All PCA-related statistics except for parallel analysis was conducted using SPSS v.24 (IBM; Armonk, NY, USA). An online engine was used for parallel analysis [16].

For univariate analysis, a Mann Whitney *U* test for the cytokine score, individual cytokine concentrations and continuous risk factors was used against MACE, while a Chi square test was used for categorical risk factors. From this test, any individual cytokine concentrations that were significantly different between patients with and without MACE were combined into a score using principal component analysis. A Mann Whitney *U* test was also conducted on this subset score. Receiver operator characteristic (ROC) curves were generated and multinomial logistic regression was used for any PCA-derived scores that were statistically significant on univariate analysis. SPSS v.24 was used to complete these analyses.

Finally, to conduct a post-hoc power calculation, a student’s T test was used to compare the means and standard deviations of the cytokine score in patients who did and did not develop MACE (G*Power v.3.0.10, University of Düsseldorf; Germany). A post-hoc power calculation showed that the cohort had 62.6% power to detect a significant difference in means of cytokine scores between patients who did and did not develop MACE (significance calculated by α = 0.05).

## 3 Results

### 3.1 Baseline demographics

From a population of 1994 patients in the biobank, 320 patients met the study criteria and were included in the cohort. Reasons for exclusion included blood samples not collected within 48–72 h from symptom onset (1581, 94.4%), treatment with thrombolysis (33, 1.97%), renal insufficiency (26, 1.55%), subsequent revision to an alternative diagnosis from AMI (18, 1.08%), and other factors such as no plasma samples collected and undetermined time of symptom onset (16, 0.96%). A further three patients were excluded due to loss to follow-up, resulting in a final cohort of 317 patients. Table 1 presents the baseline demographics of the cohort. Of the 317 patients, 76.0% were male, 83.0% were European and 78.9% were diagnosed with NSTEMI. The mean age was 62.5 years. From this cohort, 41 (12.9%) patients developed MACE within one year of follow-up, which comprised of four deaths, 16 AMIs, seven ischaemic strokes, one stent thrombosis, eight CHFs and five unplanned revascularisations. We observed that patients who developed MACE were older (66.6 versus 61.9 years old, p-value = 0.022), more likely to present with STEMI (36.6% versus 18.8%, p-value = 0.014) and more likely to have a previous history of stroke or TIA (14.6% versus 4.70%, p-value = 0.024).

**Table 1:** Baseline demographics.

**Demographics**	**Total (N = 317)**	**MACE (n = 41)**	**No MACE (n = 276)**	**P-value**
Male, n (%)	241 (76.0)	29 (70.7)	212 (76.8)	0.433
Age, mean years (SD)	62.5 (11.0)	66.6 (11.0)	61.9 (10.9)	**0.022**
BMI, median (IQR)^1^	28.9 (5.97)	30.3 (5.44)	28.4 (6.06)	0.087
Ethnicity, n (%):				
*European*	263 (83.0)	34 (82.9)	229 (83.0)	0.887
*Māori and Pasifika people*	35 (11.0)	4 (9.80)	31 (11.2)	
*Other*	19 (5.99)	3 (7.30)	16 (5.80)	
**Risk Factors**				
Prior MI, n (%)	80 (25.2)	11 (26.8)	69 (25.0)	0.848
HTN, n (%)	177 (55.8)	24 (58.5)	153 (55.4)	0.739
Diabetes, n (%)	56 (17.4)	10 (24.4)	45 (16.3)	0.267
Dyslipidaemia, n (%)	179 (56.5)	24 (58.5)	155 (56.2)	0.866
Family history of CAD, n (%)	121 (38.2)	16 (39.0)	105 (38.0)	1.00
CHF (Kilip Class 2 or 3)	4 (1.30)	0 (0.00)	4 (1.40)	0.656
AF, n (%)	22 (6.88)	5 (12.2)	17 (6.20)	0.181
Stroke/TIA, n (%)	19 (5.94)	6 (14.6)	13 (4.70)	**0.024**
Smoking, n (%):				
*Current*	67 (21.1)	6 (14.6)	61 (22.1)	0.585
*Former*	113 (35.6)	16 (39.0)	97 (35.1)	
*Never*	137 (43.2)	19 (46.3)	118 (42.8)	
Peak TnT, median (IQR)^2^	519 (1399)	949 (2834)	496 (1229)	0.145
**Clinical Presentation**				
STEMI, n (%)	67 (21.1)	15 (36.6)	52 (18.8)	**0.014**
NSTEMI, n (%)	250 (78.9)	26 (63.4)	224 (81.2)	

Continuous, parametric variables of the total cohort are expressed as mean (SD) while continuous, non-parametric variables are expressed as median (IQR). Categorical variables are expressed as frequency (percentage). Significant p-values (p < 0.05), calculated using the exact significance two-sided Chi-square test, are bolded.

1 Calculated from N = 315 in the total population, with n = 274 in the no MACE population, as two patients had missing BMI values.

2 Calculated from N = 314 in the total population, with n = 273 in the no MACE population, as three patients did not have troponin T results available.

### 3.2 Descriptive statistics of individual cytokine concentrations

**Fig. 1:**
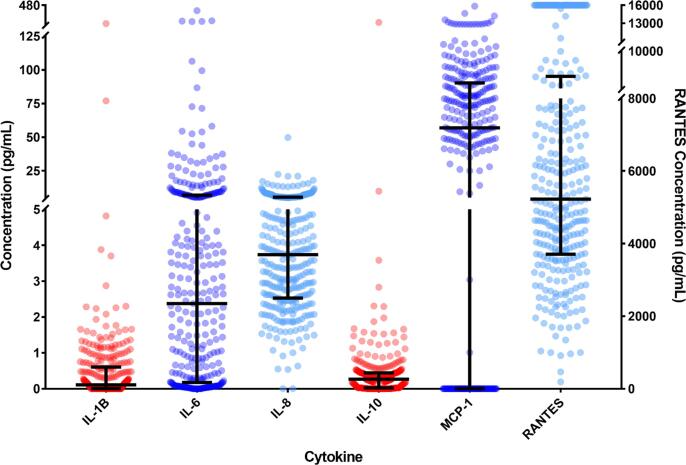
Cytokine concentrations in AMI patients measured in plasma 48 to 72 h from symptom onset. The concentrations of all cytokines shown on the left y-axis, except for RANTES, which is demonstrated on the right. For RANTES, values above the upper limit of detection were given an arbitrary value of 16,000 pg/mL. The bars represent the median and IQRs of each cytokine.

The percentage of detectable levels of the 13 cytokines within the patient population ranged from 0% to 99.4%. Cytokines with less than 50% of the patients having detectable levels (IFNγ, IL-4, IL-17A, GM-CSF, TGF-β1, TNFα, and VEGF) were excluded from further analyses. The concentrations, medians and interquartile ranges (IQRs) for the remaining six cytokines (IL-1β, IL-6, IL-8, IL-10, MCP-1, and RANTES) were plotted in Fig. 1. The medians and IQRs were calculated and plotted, revealing large IQRs and demonstrating a considerable variation in concentrations. These values can be found in Supplementary Table 2.

### 3.3 Principal component analysis

Barlett’s test of sphericity was statistically significant (p < 0.001), indicating that the cytokines were sufficiently correlated for PCA to be useful [17]. Parallel analysis revealed that only the first two components, with eigenvalues of 1.562 and 1.156, were significant. These two components accounted for 45.3% of the variance in the data and the scores of the two components were added to create the cytokine score.

Table 2 describes the correlations of each cytokine in relation to the loading for each component. IL-6, IL-8 and IL-10 had large correlations for the first component (r = 0.768, 0.722 and 0.536, respectively), while MCP-1 and IL-10 had the greatest correlations for the second component (r = 0.638 and −0.534, respectively), suggesting they have the greatest contributions to the loading of the components.

**Table 2:** Component matrix demonstrating correlations between the first and second components for each cytokine.

**Cytokine**	**Correlation (First component)**	**Correlation (Second component)**
IL-1β	−0.031	0.247
IL-6	0.768	−0.358
IL-8	0.722	0.378
IL-10	0.536	−0.534
MCP-1	0.371	0.638
RANTES	0.160	0.364

Correlations less than −0.5 or >0.5 demonstrate a significant loading or contribution to the variance in the component.

### 3.4 Predictors of MACE – Univariate analysis

**Fig. 2:**
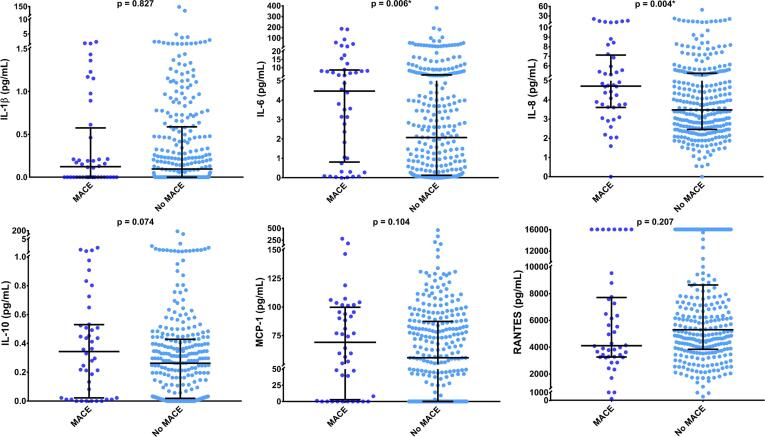
Distributions of individual cytokines in patients with and without MACE. The medians and IQRs of the six cytokines used to create the cytokine score were compared in patients with and without MACE using the Mann Whitney *U* test. Only IL-6 and IL-8 concentrations were significantly higher in those who developed MACE. * = Significant p-value (p < 0.05).

**Fig. 3:**
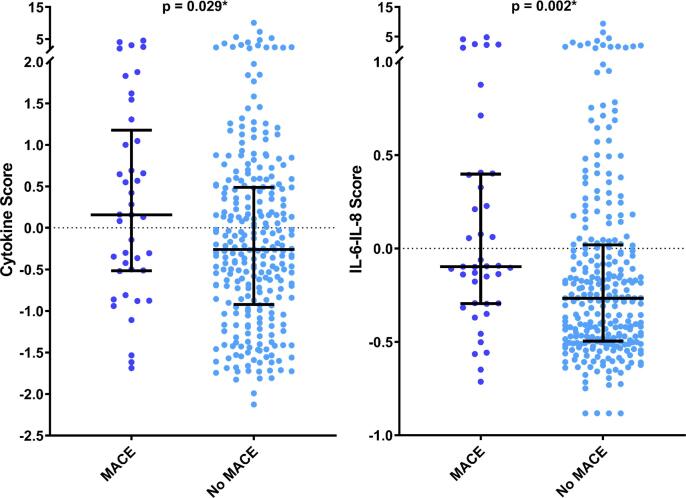
Distributions of PCA-derived scores in patients with and without MACE. The medians and IQRs of the cytokine score and IL-6-IL-8 score were compared in patients with and without MACE, using Mann-Whitney *U* test. Both scores were significantly higher in those who developed MACE at one year. * = Significant p-values (p < 0.05).

Fig. 2, Fig. 3 display the medians and IQRs for the individual cytokines and PCA-derived cytokine scores in patients who did and did not develop MACE at one year. Supplementary Table 3 presents the information in Fig. 2, Fig. 3. As IL-6 and IL-8 were the only cytokines that were statistically significantly different between the two cohorts (p = 0.006 and p = 0.004, respectively), PCA was used to create an IL-6-IL-8 score. The Bartlett’s test of sphericity was statistically significant (p < 0.001). Only one component was significant, with an eigenvalue of 1.347. This component accounted for 67.4% of the total variance of the two cytokines. The score of this component was used for the IL-6-IL-8 score. The medians, IQRs and p-value from the Mann Whitney *U* test were added to Supplementary Table 3. Alongside IL-6 and IL-8 levels, both the cytokine score and the IL-6-IL-8 score were statistically associated with MACE (p < 0.05).

**Fig. 4:**
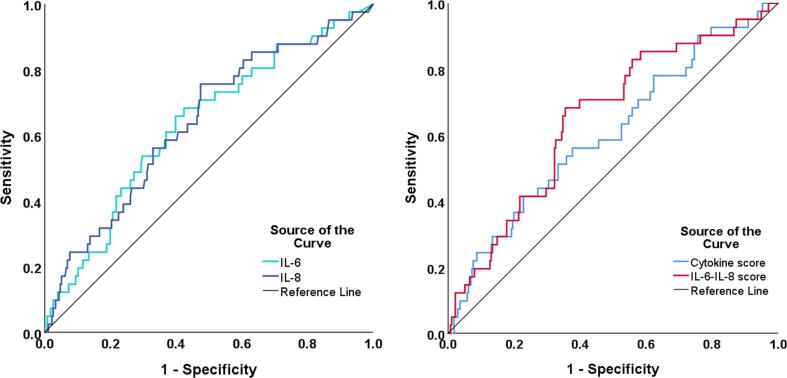
ROC curve of significant individual cytokines and PCA-derived scores as predictors of MACE. The ROC curves demonstrate sensitivity (y-axis) and 1-specificity (x-axis) for IL-6 and IL-8 individually (left), and for the cytokine score and the IL-6-IL-8 score (right), as predictors of MACE. All had a moderate AUC that was significant (p < 0.05).

To evaluate the prognostic ability of the various cytokines and PCA-derived scores for MACE, ROC curves were generated. The cytokine score and IL-6-IL-8 score were found to be moderate predictors of MACE, with significant area under the curves (AUCs) of 0.606 (p = 0.048) and 0.652 (p = 0.002), respectively. The ROC AUC for all cytokines and scores are shown in Table 3, and the optimal cut-off value, sensitivity and specificity of significant ROC AUCs are also included in the table. Only ROC curves of cytokines or scores with significant AUCs have been shown in Fig. 4.

**Table 3:** Area under the curve (AUC) generated from ROC curve analysis of MACE.

Cytokine/Score	ROC AUC	Cut-off Value	Sensitivity (%)	Specificity (%)
IL-1β	0.490	–	–	–
IL-6	**0.632**	3.11 pg/mL	65.9	60.1
IL-8	**0.639**	3.59 pg/mL	75.6	52.5
IL-10	0.586	–	–	–
MCP-1	0.578	–	–	–
RANTES	0.439	–	–	–
Cytokine score	**0.606**	0.0802	56.1	62.3
IL-6-IL-8 score	**0.652**	−0.141	65.9	65.2

All significant AUCs are bolded (p < 0.05). For significant AUCs, a cut-off value with optimal sensitivity and specificity has been determined from the ROC curve, with sensitivity prioritised over specificity when deciding between two similar cut-offs.

### 3.5 Predictors of MACE – multivariate analysis

As all individual cytokines were used to derive the cytokine score, and IL-6 and IL-8 individually were used to derive the IL-6-IL-8 score, these could not be combined into one multinomial logistic regression model. Therefore, cytokine scores above 0.0802, age and clinical presentation (the baseline characteristics that were statistically significant on univariate analysis) were combined into one multivariate model (Model 1); IL-6-IL-8 scores above −0.141 and clinical factors were combined into Model 2; and IL-6 levels above 3.11 pg/mL, IL-8 levels above 3.59 pg/mL, and clinical factors were combined into Model 3. With only 19 patients (6.0%) in the cohort having a previous history of stroke or TIA, this variable was excluded from all models. Clinical presentation remained significantly associated with MACE in all three models and age was significant in the first model. Of the two PCA-derived scores, only the IL-6-IL-8 score was found to be an independent predictor of MACE at one year, with an OR of 2.77 (95% CI 1.32–5.81), p = 0.007 (Table 4). IL-6 alone was also an independent predictor of MACE (OR 2.18, CI 1.06–4.50, p = 0.035).

**Table 4:** Multivariate models of MACE.

**Risk Factor**	**Model 1 OR (95% CI)**	**P-value**	**Model 2 OR (95% CI)**	**P-value**	**Model 3 OR (95% CI)**	**P-value**
Age	1.04 (1.01–1.07)	**0.026**	1.03 (0.992–1.06)	0.139	1.03 (0.992–1.06)	0.131
STEMI	2.58 (1.25–5.29)	**0.010**	2.24 (1.08–4.65)	**0.031**	2.46 (1.20–5.08)	**0.026**
Cytokine score > 0.0802	3.18 (0.724–13.9)	0.126	–	–	–	–
IL-6-IL-8 score > −0.141	–	–	2.77 (1.32–5.81)	**0.007**	–	–
IL-6 > 3.11 pg/mL	–	–	–	–	2.18 (1.06–4.50)	**0.035**
IL-8 > 3.59 pg/mL	–	–	–	–	2.18 (0.956–4.99)	0.414

Model 1 = Age, STEMI and cytokine score>0.0802; Model 2 = Age, STEMI and IL-6-IL-8 score greater than −0.141; and Model 3 = Age, STEMI, IL-6 concentration>3.11 pg/mL and IL-8 concentration>3.59 pg/mL. Significant p-values (<0.05) are bolded.

## 4 Discussion

In this study, we created two PCA-derived scores that allowed us to mathematically reduce the data while accounting for collinearity between cytokines with overlapping functions and maximising data variance. On univariate analysis, the cytokine PCA-derived score, the IL-6-IL-8 PCA-derived score, and levels of both IL-6 and IL-8, were associated with MACE along with clinical presentation and age. ROC analysis showed modest predictive power for each of these continuous variables. Using cut-off points from that analysis, multivariate models found that either IL-6-IL-8 PCA-derived score or the IL-6 alone were independent predictors of MACE.

Combining the six cytokines where values could be calculated for > 50% of the population in the PCA model produced an AUC of 0.606 on ROC analysis, suggesting moderate ability to predict MACE. In the multivariate model, the point estimate for the ORs was 3.77, but the 95% CI crossed 1.0, such that this score was not an independent predictor of MACE. Despite this, the inclusion of multiple cytokines that have been previously linked to MACE following ACS may have merit. From a theoretical point of view, inclusion of multiple markers using a mathematical technique that deals with inter-correlations and is robust even in the presence of missing data-points is an appealing approach to characterise inflammation.

The alternative approach is to selectively include cytokines into the PCA model that are univariate predictors of outcome in a given cohort. In this instance, inclusion of IL-6 and IL-8 alone generated a PCA score that had a greater AUC of 0.652 on ROC analysis than either cytokine individually or the combined cytokine score. In addition, the IL-6-IL-8 score was an independent predictor of outcome in the multivariate model, with a greater odds ratio than either cytokine alone. This is consistent with the idea that inclusion of more than one inflammatory marker into a score may be more predictive than use of a single marker [9]. Skau et al. showed similar results in their study of AMI patients where selectively combining growth differentiation factor 15 (GDF-15) and tumour necrosis factor-related apoptosis-inducing ligand receptor 2 (TRAIL-R2) produced the same ROC AUC of 0.85 for MACE as the combination of 33 biomarkers [8].

IL-6 is expressed by many cell types, predominantly acts in a pro-inflammatory manner by mediating the acute phase response and has been associated with MACE in patients with acute coronary syndrome [4]. IL-8 is also expressed by various cells and has both pro- and anti-inflammatory roles. It is activated during myocardial ischaemic-reperfusion injury but also helps with angiogenesis, which improves myocardial function after AMI [4]. Interestingly, most studies have shown that high levels of IL-8 are associated with increased risk of adverse outcomes, but Velasquez et al. found it was protective in women [4], [18], [19], [20]. To the best of our knowledge, no previous study has looked at combining IL-6 and IL-8 alone to predict MACE in AMI. This is likely because both cytokines have pro-inflammatory actions, making them unsuitable to be combined into a ratio as this type of analysis is normally reserved for two cytokines with opposing roles in inflammation. Moreover, these two cytokines are often highly correlated, so if they were to be included in a simple score that did not control for their overlapping functions, the inflammatory pathways associated with these cytokines would be over-represented in the score. This makes it difficult to derive meaningful scores from both sets of data. However, as demonstrated by this study, PCA can be useful for combining these two cytokines as it takes into account the collinearity that exists between them.

Few studies have investigated PCA-derived scores as predictors of MACE following AMI. One study analysed 12 biomarkers in 100 STEMI patients and used exploratory factor analysis (EFA), a statistical method similar to PCA, to create factor scores and correlated these with 30-day MACE [21]. Only two factor scores, one of which was composed of IL-6, IL-8 and MCP-1, were found to be independent predictors. With our cohort, IL-6 and IL-8 contributed to the first component score, while MCP-1 contributed to the second component score. The differences in our findings may be due to the rotational component of EFA, which allows for improved clustering of variables than PCA while compromising the amount of variance retained by the model [22]. In this study, we prioritised retaining the maximum amount of variance possible in our cytokines over correlations between cytokines, as there was large variance in the individual cytokines concentrations among AMI patients, thus we chose PCA over EFA.

Each of the cytokines chosen for analysis has been associated with adverse outcomes following ACS in at least one prior study (Supplementary Table 1). This was the basis for inclusion of these markers in the current study. However, there are many other inflammatory cytokines which have been used to predict outcome following ACS [7], [8] that were not able to be included in this study due to limited resources. Additionally, six of the 13 cytokines analysed were undetectable in our cohort. This may be due to using a sampling time that was too distant from symptom onset. To standardise timing in this study, we chose to include patients where blood was collected 48–72 h after symptom onset, as this was the most frequent time period in our biobank, allowing us to examine these trends in a larger cohort of patients. While this limited our ability to analyse only seven markers, the combination and sub-combination were still predictive of MACE. IL-1β may peak significantly sooner than 48 h [23], and other markers, including IL-6, MCP-1 and IFNγ, have been shown to have dynamic changes in concentration over time [24], [25]. The optimal time point for measuring peak levels of cytokines in the context of ACS has not been determined. Further investigation is required regarding the temporal changes in concentrations for all cytokines and how this might affect a cytokine score.

## 5 Conclusion

Use of PCA to derive an inflammatory score from multiple cytokines was predictive of MACE in AMI patients, with the best model achieved by inclusion of only those cytokines that were individually associated with MACE. In this study, these cytokines were IL-6 and IL-8. The PCA-derived score had moderate sensitivity and specificity. While this approach appears promising, the optimal set of cytokines to measure, and the optimal time to measure them remains to be determined.

**Source of funding**

This study was not funded by any specific grants and used departmental funding from the 10.13039/100008247University of Otago Wellington, New Zealand.

## CRediT authorship contribution statement

**Gisela A. Kristono:** Methodology, Formal analysis, Investigation, Writing - original draft, Writing - review & editing, Visualization. **Ana S. Holley:** Conceptualization, Methodology, Investigation, Resources, Writing - review & editing, Supervision. **Kathryn E. Hally:** Investigation, Resources, Writing - review & editing. **Morgane M. Brunton-O’Sullivan:** Investigation, Writing - review & editing. **Bijia Shi:** Methodology. **Scott A. Harding:** Conceptualization, Writing - review & editing, Supervision. **Peter D. Larsen:** Conceptualization, Methodology, Writing - review & editing, Supervision, Funding acquisition.

## Declaration of Competing Interest

The authors declare that they have no known competing financial interests or personal relationships that could have appeared to influence the work reported in this paper.
