# Supplementary material for: An IL-6-IL-8 score derived from principal component analysis is predictive of adverse outcome in acute myocardial infarction
Source: Cytokine X. 2020 Oct 8;2(4):100037. doi: 10.1016/j.cytox.2020.100037 (PMC7885891; doi:10.1016/j.cytox.2020.100037)
Supplement: Supplementary data 1 [file mmc1.docx]

# Supplementary material for An IL-6-IL-8 score derived from principal component analysis is predictive of adverse outcome in acute myocardial infarction

1. Rationale for chosen cytokines, method of analysis, and concentrations of lowest standard detected

| **Cytokine** | **Assay** | **Lowest standard** | **Rationale** |
| --- | --- | --- | --- |
| IL-1β | ELISA | 0.497 | Indicated as a prognostic marker for in-hospital outcomes in AMI and associated with LV dysfunction in AMI.(1-3) Inhibition of IL-1β with canakinumab showed reduced MACE (non-fatal MI, cardiovascular death or stroke) in stable CAD.(4) |
| IL-4 | CBA | 0.274 | Indicated as a prognostic marker for severe LV dysfunction in STEMI.(5) |
| IL-6 | CBA | 0.274 | Indicated as a prognostic marker for MACE, death or HF in ACS.(6-8) |
| IL-8 | CBA | 0.274 | Indicated as a prognostic marker for MACE and restenosis in AMI.(9,10) |
| IL-10 | CBA | 0.274 | Associated with MACE and impaired LVEF in AMI, and death in STEMI.(8,11,12) |
| IL-17A | CBA | 0.274 | Indicated as a prognostic marker for MACE and restenosis in AMI.(10,13) |
| GM-CSF | CBA | 10.0 | Indicated as a prognostic marker for LV dysfunction.(14) However, GM-CSF treatment in STEMI patients was associated with improved LVEF.(15,16) |
| IFNγ | CBA | 0.274 | Indicated as a prognostic marker for restenosis in AMI and severe LV dysfunction in STEMI.(5,10) |
| MCP-1 | CBA | 10.0 | Indicated as a prognostic marker for MACE, death and non-fatal MI in ACS.(17,18) |
| RANTES | CBA | 10.0 | Indicated as a prognostic marker for mortality and progressive atherosclerosis in ACS.(19,20) |
| TGF-β1 | CBA | 19.5 | Associated with LVEF in STEMI.(21) |
| TNFα | CBA | 0.274 | Indicated as a prognostic marker for MACE in STEMI and ACS.(22,23) |
| VEGF | CBA | 10.0 | Indicated as a prognostic marker for MACE in ACS.(24,25) |

The assay used to detect the cytokine, the concentration of the lowest standard and the rationale behind the cytokines chosen for this study are listed above. All concentrations are in pg/mL and are rounded to three significant figures. ELISA = Enzyme-linked immunosorbent assay, CBA = Cytometric bead array, LV = left ventricular, CAD = coronary artery disease, LVEF = left ventricular ejection fraction. AS = acute coronary syndrome, HF = heart failure.

1. **Descriptive Statistics of Individual Cytokines**

| **Cytokine** | **n (%)** | **Median (IQR)** |
| --- | --- | --- |
| IL-1β | 180 (56.8) | 0.108 (0.606) |
| IL-6 | 304 (95.9) | 2.38 (6.72) |
| IL-8 | 314 (99.1) | 3.74 (2.82) |
| IL-10 | 271 (85.5) | 0.264 (0.422) |
| MCP-1 | 217 (68.5) | 56.9 (90.3) |
| RANTES | 253 (79.8) | 5224 (4949) |
| IFNγ | 0 (0.00) | - |
| IL-4 | 19 (5.99) | - |
| IL-17A | 53 (16.7) | - |
| GM-CSF | 17 (5.36) | - |
| TGF-β1 | 2 (0.631) | - |
| TNFα | 10 (3.15) | - |
| VEGF | 108 (34.1) | - |

The frequency (n) and percentage of patients with detectable concentrations have been calculated for each cytokine. The medians and IQRs (pg/mL) were not calculated for cytokines where <50% of patients had detectable levels. N = 317.

1. Univariate analysis of inflammation and MACE

| **Cytokine** | **MACE** | **No MACE** | **P-value** |
| --- | --- | --- | --- |
| IL-1β | 0.130 (0.750) | 0.093 (0.600) | 0.827 |
| IL-6 | 4.55 (9.67) | 2.08 (5.86) | **0.006** |
| IL-8 | 4.74 (4.37) | 3.48 (2.81) | **0.004** |
| IL-10 | 0.358 (0.540) | 0.263 (0.410) | 0.074 |
| MCP-1 | 76.2 (96.9) | 55.6 (87.8) | 0.104 |
| RANTES | 4128 (4987) | 5355 (4809) | 0.207 |
| Cytokine score | 0.156 (1.69) | -0.260 (1.41) | **0.029** |
| IL-6-IL-8 score | -0.098 (0.693) | -0.267 (0.515) | **0.002** |

Median (IQRs) of individual cytokines, the PCA-derived combined cytokine score and the PCA-derived IL-6-IL-8 score are calculated in MACE and non-MACE patients. The p-values are generated from Mann-Whitney U tests and significant p-values (p <0.05) are bolded.

## References

1. Orn S, Ueland T, Manhenke C, Sandanger O, Godang K, Yndestad A, et al. Increased interleukin-1beta levels are associated with left ventricular hypertrophy and remodelling following acute ST segment elevation myocardial infarction treated by primary percutaneous coronary intervention. Journal of Internal Medicine. 2012;272(3):267-76.

2. Correia LC, Andrade BB, Borges VM, Clarencio J, Bittencourt AP, Freitas R, et al. Prognostic value of cytokines and chemokines in addition to the GRACE Score in non-ST-elevation acute coronary syndromes. Clinica chimica acta; international journal of clinical chemistry. 2010;411(7-8):540-5.

3. Blum A, Sclarovsky S, Rehavia E, Shohat B. Levels of T-lymphocyte subpopulations, interleukin-1β, and soluble interleukin-2 receptor in acute myocardial infarction. American Heart Journal. 1994;127(5):1226-30.

4. Ridker PM, Everett BM, Thuren T, MacFadyen JG, Chang WH, Ballantyne C, et al. Antiinflammatory Therapy with Canakinumab for Atherosclerotic Disease. New England Journal of Medicine. 2017;377(12):1119-31.

5. Szkodzinski J, Hudzik B, Osuch M, Romanowski W, Szygula-jurkiewicz B, Polonski L, et al. Serum concentrations of interleukin-4 and interferon-gamma in relation to severe left ventricular dysfunction in patients with acute myocardial infarction undergoing percutaneous coronary intervention. Heart and vessels. 2011;26(4):399-407.

6. Fanola CL, Morrow DA, Cannon CP, Jarolim P, Lukas MA, Bode C, et al. Interleukin‐6 and the Risk of Adverse Outcomes in Patients After an Acute Coronary Syndrome: Observations From the SOLID‐TIMI 52 (Stabilization of Plaque Using Darapladib—Thrombolysis in Myocardial Infarction 52) Trial. Journal of the American Heart Association: Cardiovascular and Cerebrovascular Disease. 2017;6(10):e005637.

7. Lopez-Cuenca A, Manzano-Fernandez S, Lip GYH, Casas T, Sanchez-Martinez M, Mateo-Martinez A, et al. Interleukin-6 and high-sensitivity c-reactive protein for the prediction of outcomes in non-ST-segment elevation acute coronary syndromes. [Spanish]. Revista Espanola de Cardiologia. 2013;66(3):185-92.

8. Ammirati E, Cannistraci CV, Cristell NA, Vecchio V, Palini AG, Tornvall P, et al. Identification and predictive value of interleukin-6+ interleukin-10+ and interleukin-6- interleukin-10+ cytokine patterns in ST-elevation acute myocardial infarction. Circ Res. 2012;111(10):1336-48.

9. Shetelig C, Limalanathan S, Hoffmann P, Seljeflot I, Gran JM, Eritsland J, et al. Association of IL-8 With Infarct Size and Clinical Outcomes in Patients With STEMI. Journal of the American College of Cardiology. 2018;72(2):187-98.

10. Karaulov Alexandr V, Markelova Elena V, Grachev Nikita I, Semenikhin Andrey A, Glushkova Natalya E. Immune biomarkers of percutaneous coronary intervention adverse outcomes in myocardial infarction and stable angina patients. Asian J Pharm. 2018;12(3):S1069-S75.

11. Novo G, Bellia C, Fiore M, Bonomo V, Pugliesi M, Giovino M, et al. A Risk Score Derived from the Analysis of a Cluster of 27 Serum Inflammatory Cytokines to Predict Long Term Outcome in Patients with Acute Myocardial Infarction: a Pilot Study. Ann Clin Lab Sci. 2015;45(4):382-90.

12. Lakhani HV, Khanal T, Gabi A, Yousef G, Alam MB, Sharma D, et al. Developing a panel of biomarkers and miRNA in patients with myocardial infarction for early intervention strategies of heart failure in West Virginian population. PLoS ONE. 2018;13(10):e0205329.

13. Simon T, Taleb S, Danchin N, Laurans L, Rousseau B, Cattan S, et al. Circulating levels of interleukin-17 and cardiovascular outcomes in patients with acute myocardial infarction. European Heart Journal. 2013;34(8):570-7.

14. Parissis JT, Adamopoulos S, Venetsanou K, Kostakis G, Rigas A, Karas SM, et al. Plasma profiles of circulating granulocyte-macrophage colony-stimulating factor and soluble cellular adhesion molecules in acute myocardial infarction. Contribution to post-infarction left ventricular dysfunction. European Cytokine Network. 2004;15(2):139-44.

15. Yang C, Deng Z, Jiang X, Han K, Zhang T, Zhu W, et al. Long-term effects of primary early granulo-monocyte colony-stimulating factor treatment on the left ventricular function and remodeling in patients with acute myocardial infarction with five-year follow-up. International Journal of Cardiology. 2012;155(2):287-90.

16. Deng Z, Yang C, Deng H, Yang A, Geng T, Chen X, et al. Effects of GM-CSF on the stem cells mobilization and plasma C-reactive protein levels in patients with acute myocardial infarction. International Journal of Cardiology. 2006;113(1):92-6.

17. Tuñón J, Blanco-Colio L, Cristóbal C, Tarín N, Higueras J, Huelmos A, et al. Usefulness of a Combination of Monocyte Chemoattractant Protein-1, Galectin-3, and N-Terminal Probrain Natriuretic Peptide to Predict Cardiovascular Events in Patients With Coronary Artery Disease. The American Journal of Cardiology. 2014;113(3):434-40.

18. de Lemos JA, Morrow DA, Sabatine MS, Murphy SA, Gibson CM, Antman EM, et al. Association Between Plasma Levels of Monocyte Chemoattractant Protein-1 and Long-Term Clinical Outcomes in Patients With Acute Coronary Syndromes. Circulation. 2003;107(5):690.

19. Blanchet X, Cesarek K, Brandt J, Herwald H, Teupser D, Küchenhoff H, et al. Inflammatory role and prognostic value of platelet chemokines in acute coronary syndrome. Thromb Haemost. 2014;112(12):1277-87.

20. de Jager SCA, Bongaerts BWC, Weber M, Kraaijeveld AO, Rousch M, Dimmeler S, et al. Chemokines CCL3/MIP1α, CCL5/RANTES and CCL18/PARC are Independent Risk Predictors of Short-Term Mortality in Patients with Acute Coronary Syndromes. PLoS ONE. 2012;7(9):e45804.

21. Talasaz AH, Khalili H, Jenab Y, Salarifar M, Broumand MA, Darabi F. N-Acetylcysteine effects on transforming growth factor-β and tumor necrosis factor-α serum levels as pro-fibrotic and inflammatory biomarkers in patients following ST-segment elevation myocardial infarction. Drugs in R&D. 2013;13(3):199-205.

22. van Diepen S, Alemayehu WG, Zheng Y, Theroux P, Newby LK, Mahaffey KW, et al. Temporal changes in biomarkers and their relationships to reperfusion and to clinical outcomes among patients with ST segment elevation myocardial infarction. Journal of Thrombosis and Thrombolysis. 2016;42(3):376-85.

23. Tofik R, Sward P, Ekelund U, Struglics A, Torffvit O, Rippe B, et al. Plasma pro-inflammatory cytokines, IgM-uria and cardiovascular events in patients with chest pain: A comparative study. Scandinavian Journal of Clinical and Laboratory Investigation. 2015;75(8):638-45.

24. Matsudaira K, Maeda K, Okumura N, Yoshikawa D, Morita Y, Mitsuhashi H, et al. Impact of Low Levels of Vascular Endothelial Growth Factor After Myocardial Infarction on 6-Month Clinical Outcome: Results From the Nagoya Acute Myocardial Infarction Study. Circ J. 2012;76(6):1509-16.

25. Heeschen C, Dimmeler S, Hamm CW, Boersma E, Zeiher AM, Simoons ML. Prognostic Significance of Angiogenic Growth Factor Serum Levels in Patients With Acute Coronary Syndromes. Circulation. 2003;107(4):524-30.
